# The Role of Nurse Implementation Scientists in Leading Health System Transformation in Atlantic Canada and Beyond: A Discussion Paper

**DOI:** 10.1111/jan.16651

**Published:** 2024-12-06

**Authors:** Alyson Campbell, Christine Cassidy

**Affiliations:** ^1^ Faculty of Nursing University of Prince Edward Island Charlottetown Prince Edward Island Canada; ^2^ School of Nursing Dalhousie University Halifax Nova Scotia Canada

**Keywords:** health care transformation, implementation science, nurse implementation scientist

## Abstract

**Aim:**

To discuss and provide examples of how nurse implementation scientists can support health system transformation.

**Design:**

Discussion paper.

**Methods:**

Using Prince Edward Island's health system strategic plan as a case exemplar, selected key priorities in the strategic plan were mapped with examples and discussion of *how* nurse implementation scientists can support health system transformation on Prince Edward Island and beyond.

**Conclusion:**

Accelerating the development and delivery of evidence‐informed services that support health system transformation is needed. Nurse implementation scientists are ideally positioned to lead these efforts. Appropriate resourcing and compensation are essential to fully embrace nurse implementation scientist roles, collaboration, and buy‐in from health system leaders.

**Implications for Nursing:**

Practical examples of how nurse implementation scientists can lead health system transformation in a rigorous, evidence‐informed way are identified.

**Impact:**

Literature providing examples of *how* nurse implementation scientists can make meaningful impacts within health systems, particularly in rural contexts, is limited. Nurse implementation scientists are ideally positioned to collaborate with and lead health system transformation by virtue of their knowledge, skills, qualifications and experience. Implications of this work extend beyond nursing to other health disciplines, health organisations, government leaders, researchers and populations. The discussion and examples provided may be applicable to similar contexts.

**Patient or Public Contribution:**

No patient or public contribution.

**Summary**

- What does this paper contribute to the wider global community?
  ○ An embedded nurse implementation scientist is one strategy enabling nurses to lead health system transformation and sustainability.
  ○ Doctorate programmes prepare nurses to lead implementation science efforts within health systems and positions nursing to curate leaders with expertise in evidence‐informed clinical practices and application of implementation science.
  ○ Examples of how nurse implementation scientists can work within health systems may be applicable to different health systems to support the embedded nurse‐scientist role and overall health system improvement.

## 1 Introduction

Implementing evidence‐informed practices and innovations into health care systems has gained rapid global interest (Jackson et al. 2020). However, even in the presence of evidence consensus, system‐wide health care transformations are often hampered by various factors including competing policy priorities, entrenched cultural norms and mismatched resources (Sarkies et al. 2022). As a result, an estimated 80% of medical research dollars do not make a public health impact and less than 50% of clinical innovations get routinely used (Chalmers and Glasziou 2009; Jackson et al. 2020; Morris et al. 2023). Further, fewer than 40% of health care innovations successfully transition from adoption to sustained implementation (Flynn et al. 2021; Health Quality Ontario 2013; Westerlund, Sundberg, and Nilsen 2019). To address these issues, a growing field of research on how to improve health care has emerged, known as implementation science (Graham et al. 2006; Wensing and Wilson 2023). The need for implementation science research could not be greater or timelier. Health systems worldwide are under tremendous strain and must focus on how to accelerate the development and delivery of evidence‐informed services to improve and sustain health and well‐being for all (Theobald et al. 2018).

Transforming health systems requires active engagement and advocacy from a range of partners, including patients, front line care providers, health system and government leaders and researchers working in partnership to address the drivers of inequities, inefficiencies and poor patient outcomes within existing health systems (National Academies of Sciences, Engineering, and Medicine 2021). Nurses, who comprise the largest proportion of health care providers globally, are ideally positioned to lead health system transformation by virtue of their collective breadth of experience and understanding of health care contexts across sectors and populations.

## 2 Background

The global, and long‐standing challenges health care systems are facing, including crises in long‐term care, emergency departments, intensive care units, and record levels of stress, burnout and turnover among essential health care providers prevail and have only been exacerbated by the COVID‐19 pandemic (Burhans and Alligood 2010; Flynn et al. 2017; Glazier 2023; Murray et al. 2013; Statistics Canada 2022). Prince Edward Island (PEI), Canada's smallest province, is not immune to these challenges as recent reports have confirmed suggesting PEI requires a different approach to health care delivery to overcome these challenges, achieve the province's health care goals and ultimately improve population health services and outcomes (Brookins and Prince Edward Island Nurses Union 2024; Campbell and Ryan 2023; Lazarenko 2024; Morse 2023a; Ryan 2023; Spindle Strategy 2023; Walton 2023; Yarr 2024).

It has been suggested that health systems are analogous to complex organisms, where complex, dynamic relationships can support or hinder effective functioning (Jackson et al. 2020). Clear understandings of these complex relationships are crucial to implementing, adapting, evaluating and sustaining evidence‐informed practices in a health system. Indeed, many health care innovations are designed using Martin Eccles's famous ISLAGIATT maxim: ‘It seemed like a good idea at the time’ and suffer from little consideration to factors like behaviours or processes that need to be targeted for change to occur, or whether the innovation would (or even could) address any underlying factors (Wilson and Kislov 2022). To move beyond Eccles's maxim, implementation science is essential.

The knowledge and perspectives nurses have is unparalleled and essential to transforming health care systems (Cassidy, Flynn, and Shuman 2021; Flynn et al. 2017). Based on our collective experiences and expertise in clinical practice, academia and research, we argue that nurse implementation scientists have the potential to lead health care system transformation on PEI, and beyond. Using the Health PEI Strategic Plan 2021–2024 (HPEI Strategic Plan) as a case exemplar, we discuss *how* nurse implementation scientists can address key priorities that have been identified for transforming PEI's health system. Our discussion addresses a notable gap in the literature by providing tangible examples of how nurse implementation scientists can support health system transformation more broadly.

### 2.1 Implementation Science

Implementation science is the scientific study of methods to promote the systematic uptake of evidence‐informed interventions, programmes or innovations (herein referred to as ‘innovations’) into routine practice (Eccles and Mittman 2006; Wilson and Kislov 2022). The overarching aims of implementation science are threefold: (1) to describe the process of translating research into practice, (2) to understand what influences implementation outcomes and (3) to evaluate the implementation of interventions (Braithwaite, Marks, and Taylor 2014; Braithwaite et al. 2018). Implementation science explores outcomes related to the implementation process of health innovations including acceptability, appropriateness, adoption, feasibility and sustainability while also considering outcomes important to quality improvement (QI) such as safety, efficacy, equality and efficiency (Boehm, Stolldorf, and Jeffery 2020; Proctor et al. 2011) Reviews and policies worldwide have identified the need to embrace implementation science to drive health system transformation (Eccles and Mittman 2006; Koczwara et al. 2018; Leeman et al. 2021; Melder et al. 2020; Tyler and Glasgow 2021).

### 2.2 Nurse Implementation Scientists

Nurses have a rich tradition of working creatively, and thinking critically to solve problems and improve care in complex health care settings (National Academies of Sciences, Engineering, and Medicine 2021). The collective breadth of experience and understanding of the practice context among nurses is unparalleled, providing invaluable knowledge and insight important to implementation science (Cassidy, Flynn, and Shuman 2021; Flynn et al. 2017). Given nurses' unique clinical role, affinity for interprofessional collaboration and involvement with implementing evidence informed practices in clinical settings, they have a vested interest in implementation science and are excellently positioned to be leaders in meaningful health system transformations (Boehm, Stolldorf, and Jeffery 2020; Tomblin Murphy et al. 2022).

To build capacity for better implementation and cultivate leaders in implementation science, the National Implementation Research Network (NIRN) has developed a practice guide identifying competencies that implementation scientists need to support effective implementation and scaling of evidence informed innovations. These competencies reflect the necessary abilities of implementation scientists including specific knowledge, resources and skills they require. The NIRN identified 15 core competencies needed by the implementation scientist, which can be grouped into three domains: co‐creation and engagement, ongoing improvement and sustaining change (Metz et al. 2020). Table 1 provides an overview of these domains and competencies.

**TABLE 1:** National implementation research network domains, core competencies and key activities of implementation scientists.

Core competencies	Key activities
Domain 1: Co‐creation and engagement	
1. Co‐learning	Understand the system and organisational context and culture. Create spaces for new ideas to emerge. Negotiate and build trust and respect for all perspectives, including those that may be at risk of being excluded from dialogue because of race, ethnicity, language or status. Communicate and listen for the purpose of mutual understanding and the collaborative integration of different perspectives and types of knowledge. Synthesise diverse perspectives of thought and check for understanding. Conduct dynamic and interactive trainings and provide educational materials on implementation science. Seek other ways to introduce and create readiness for an implementation science informed approach that fits with existing programmes, practices and processes.
2. Brokering	Position yourself as a bridge ‘in between’ people or groups that exist in a system and are vital for the success of an implementation. Identify individuals or groups that are relevant to involve in the implementation but not yet part of it and seek to understand the root causes and contributing factors of that disconnect. Connect otherwise disconnected individuals or groups in the system by providing advice and serving as a relational resource. Develop and regularly convene implementation groups and teams with diverse stakeholders. Promote other network weaving to connect people strategically where there is a potential of mutual benefit. Source, share and translate evidence and data of relevance to involved stakeholders. Promote opportunities for stakeholders and team members to engage with others in the use of evidence and data.
3. Addressing power differentials	Position the range of service beneficiary experiences at the centre of decision‐making and implementation activities. Identify the influence that different stakeholders may have on the implementation. Pay particular attention to those with inherently greater power to influence the implementation process and those disenfranchised from the implementation process. Use facilitation techniques to make power structures visible and to protect all voices in the implementation process. Recognise and acknowledge the loss of status and authority that may be implied in an implementation process and can impede buy‐in and engagement. Seek and gain buy‐in from formal and informal leaders to include diverse expertise in team discussions. Develop an evolving ‘collective view’ or ‘shared understanding’, rather than pushing for an artificial consensus which may perpetuate existing power structures.
4. Co‐design	Work with stakeholders to build a strong fit between intervention and implementation context before moving forward with implementation efforts. Support collaborative implementation planning and co‐development of an implementation blueprint or plan involving all relevant stakeholders. Enable the co‐design of any tools, products, processes, governance structures, service models, interventions and policies related to the implementation. Promote cyclical tests of tools, products and processes among stakeholders to iteratively improve their prototypes. Support the co‐design and/or modification of specific implementation strategies based on resources and conditions present in the local context. Facilitate design‐centred activities that have the needs of people with lived experience at the centre, using collective sense‐making and negotiation.
5. Tailoring support	Regularly assess the implementation support needs and assets of different stakeholder groups. Agree on the implementation support to be made available to different stakeholder groups, sites and/or other relevant units of support. Develop a scope and sequence of virtual and onsite meetings and other activities based on the goals of those supported. Accommodate ‘ad hoc’/‘just in time’ support needs of stakeholders. Regularly assess the effectiveness of the level of your support in matching needs, goals and context of the implementation effort. Work with implementation stakeholders to select, combine and tailor their implementation strategies to meet local needs. Continuously promote the adaptability of implementation strategies used by stakeholders.
Domain 2: Ongoing improvement	
6. Assessing needs and assets	Identify the needs that different stakeholders involved in an implementation want to see met through the change process. Support the identification of readily available and potential resources and assets to be utilised and leveraged in the implementation context. Help stakeholders understand each other's perspectives and expectations regarding the area of need or opportunity. Use data‐driven inquiry methods to support ‘discovery’ processes that holistically consider need, such as assessment data, stakeholder analysis, mapping of existing services, initiative inventory, etc. Disaggregate available evidence and data and assess, consider and discuss needs that may exist for particular subpopulations (e.g., race, ethnicity, gender, socio‐economic status and geography). Engage people with lived experience in determining needs.
7. Understanding context	Involve diverse stakeholders from throughout the system—including those belonging to specific subgroups—in identifying and understanding the implications and consequences of change efforts. Use and/or conduct evidence reviews to determine relevance and fit of the proposed intervention(s)/approach(es) with identified needs and assets. Assess the contextual fit of the proposed intervention(s)/approach(es) with the values, needs, skills and resources available in the service setting, including in specific subgroups. Assess the contextual fit of the proposed intervention(s)/approach(es) with the current political, funding and organisational landscape. Continuously identify and respond to changes in the system(s) surrounding the implementation, which affect implementation. Identify and support mitigating actions to anticipate and manage risks and assumptions for the change effort (e.g., regarding resources, commitments or buy‐in; risks or loss for different stakeholders).
8. Applying and integrating implementation TMFs, strategies and approaches	Remain up to date on evidence developed through implementation research and practice. Remain up to date on knowledge about implementation frameworks, models, theories and strategies. Educate stakeholders about the current best evidence on implementation frameworks, strategies and approaches. Include all relevant stakeholders in the selection, combination and co‐design of implementation strategies and approaches. In collaboration with relevant stakeholders, assess and discuss the appropriateness of using implementation frameworks, strategies and approaches in different contexts and settings, including with particular subgroups. Support the selection, application and integration of the range of implementation frameworks, approaches, tools and resources that are best suited for the local context of service and policy settings.
9. Facilitation	Create welcoming and engaging spaces for all participants in meetings and other facilitation activities, recognising that participants may require different kinds of support to feel welcome and participate in facilitation processes. Support a communication protocol and process that facilitates interactions among stakeholders. Support the continuous and systematic identification of barriers to implementation among different stakeholders. Enable the identification of stakeholders required to develop adequate strategies for solving implementation challenges. Serve as formal and informal facilitators as determined by an analysis of the challenge and its context. Support a balance of divergent and convergent thinking among team members, depending on the type of challenge faced. For easily named and easily solved challenges (technical challenges), support stakeholders to evaluate alternatives, summarise key points, sort ideas into categories and exercise judgement. For complex challenges with no easy solution (adaptive challenges), support stakeholders to generate alternatives, free flow open discussion, gather diverse points of view and suspend judgement. Ensure that the facilitation method used matches the challenge in focus (e.g., structured facilitation, free flowing group discussions, etc.). When needed, respond to emergent discussions or challenges with ad hoc, nimble facilitation.
10. Communication	Work with stakeholders to develop communication protocols designed to intentionally engage stakeholders, communicate progress and celebrate implementation success, report systemic barriers that are preventing or hindering implementation, report on actions taken to resolve or address implementation challenges, revisit past decisions and agreements periodically to ensure that solutions are still appropriate. Support the development of different communication protocols for different target groups, which specify communication goals, the method and frequency of communication, who needs to communicate, how effectiveness of communication will be measured and how communication will consistently be improved. Recognise and respond to differences in communication needs across different stakeholders involved, for example, due to different organisational roles, implementation expectations, involvement and responsibilities. Help implementation stakeholders to recognise and respond to differences in communication needs among focus populations through the implementation. These differences may be due to, for example, varying levels of language proficiency and literacy, different gender‐, education‐ or culture‐based norms and preferences, etc. Encourage stakeholders to regularly communicate with and gather feedback from actors within and outside the implementing system to understand how implementation processes are perceived. As part of this process, support effective communication and feedback loops among practice, supervision, management and leadership levels of the system (i.e., vertical feedback loops that support communication up and down a system), support effective communication and feedback loops among service partners, advocacy groups, training networks, representatives from the focus population and other collaborators (i.e., horizontal feedback loops that support communication across system sectors). Help to identify local communication barriers or complications and work with relevant implementation stakeholders to resolve these challenges
11. Conducting improvement cycles	Facilitate the ongoing testing and improvement of tools, products, processes, governance structures, service models and policies of relevance to implementation efforts. Help to identify and gather relevant quantitative and qualitative data about the progress and quality of implementation activities and outcomes. Ensure that different stakeholders have access to relevant, valid and reliable data on how the programme or practice and accompanying implementation strategies are functioning to guide decision‐making along the way. Support the development of processes and structures for the routine collection and analysis of these data. Promote the collection and use of data suitable to understand the differential impact of interventions and their implementation on different focus populations and communities. Develop stakeholders' capacity to continuously assess and use data for decision‐making about the ongoing planning, implementation and outcomes of a programme or practice through modelling, instruction and coaching. Support the development of structures that ensure that stakeholders regularly dedicate time to reflecting on or debriefing about available data throughout an implementation as a strategy to promote shared learning and improvements along the way. Support implementation stakeholders in their data‐based decision‐making, including the prioritisation of needs and challenges, and the development of concrete solutions to identified problems. Help to create feedback loops that connect leadership with front line service, and policy with practice and/or research and ensure that improvements made during implementation are communicated to all stakeholders.
Domain 3: Sustaining change	
12. Growing and sustaining relationships	Build trust with others by modelling transparent action and accountability. Seek out relationships with implementation stakeholders from all aspects of the system. Engage in ongoing self‐assessment and diagnostic assessment of relationship strengths and weaknesses. Encourage and make use of feedback to strengthen relationships. Regulate distress in relationships by creating space for stakeholders to discuss challenges and dispute assumptions when conflict emerges. Seek to demonstrate value to the stakeholders involved in implementing. Enter the implementation space with humility as a learner, recognising that local actors have important expertise and experience to contribute to the implementation process. Demonstrate commitment and persistence in the face of complex challenges. Have difficult conversations with stakeholders and be open to feedback. Show kindness and vulnerability. Demonstrate empathy.
13. Developing teams	Help to identify relevant implementation team members among those involved in the implementation. Facilitate the development of clear governance structures for implementation teams, for example, through team charters or agreements. Support the development of effective team meeting processes including the establishment of consistent meeting schedules and standing agendas. Ensure teams have support from leadership for their roles and functions. Help to develop communication protocols to support feedback loops among multiple, linked implementation teams for a single initiative. Develop processes for the continuous assessment and improvement of team functioning, including gathering feedback from team members. Ensure that implementation teams provide opportunities for members to learn and grow through participation on the team. Work to enhance team cohesion and trust among team members. Help to manage conflict among team members.
14. Building capacity	Prepare stakeholders for necessary changes, model or simulate the change that will be implemented prior to implementation. Work with stakeholders to assess capacity strengths and needs related to the implementation. Provide or secure training needed for stakeholders and partners to gain capacity, and connect with others who can provide training, modelling and coaching. Model the use of knowledge, skills, behaviours, attitudes and practices for stakeholders to demonstrate application in a real‐world setting. Coach stakeholders' use of knowledge, skills, behaviours, attitudes and practices in their daily work so that partners can gain confidence and competency. Identify and help to change and/or implement organisational processes and structures required to develop capacity for implementation (e.g., human resources, technology). Support stakeholders in identifying potential future external and internal challenges to implementation (related to, e.g., finances, policies or staffing) and to develop strategies for building sufficient capacity to meet these. Promote collaboration and partnerships to build and expand capacities (e.g., through the use of community–academic partnerships, learning collaboratives, etc.).
15. Cultivating leaders and champions	Identify existing leadership roles of relevance to implementation efforts. Work and communicate with relevant formal leaders involved in the implementation to define, understand and develop their role and capacity as implementation leaders (e.g., through the use of appreciative inquiry or reflection techniques). Develop processes and structures for regularly debriefs with leaders central to implementation efforts. Support leaders to access data and information required for them to stay abreast of implementation work and the decisions they should be involved in. Identify emerging leaders in or around the implementing system and consider how they may be further involved in and developed through implementation efforts (e.g., through the use of power analysis or systems mapping tools). Support emerging leaders to share responsibilities and develop their confidence and competency (e.g., by co‐facilitating meetings, co‐supervising staff, etc.). When leadership transitions occur, work with stakeholders to provide planning, continuity, analysis and support as needed to ease the transition. In collaboration with implementation stakeholders, identify champions within and outside the implementing system with a potential to positively influence the implementation efforts. Support stakeholders in developing strategies to contact and engage these champions in relevant ways throughout the course of the implementation. Ensure stakeholders regularly review championship roles to assess whether these are being cultivated as intended, are operating as desired, or if any losses in championship have occurred. If challenges are identified, facilitate problem solving as needed.

*Note:* Adapted from Metz, A., Burke, K., Albers, B., Louison, L., & Bartley, L. (2020, December). A practice guide to supporting implementation. What competencies do we need? National Implementation Research Network. https://nirn.fpg.unc.edu/sites/nirn.fpg.unc.edu/files/resources/ISP%20Practice%20Guide‐v1‐10.27.22.pdf.

Specific activities of implementation scientists in achieving these competencies will be unique to each health care system and dependent on contextual factors including leadership and governance, products and services, needs and priorities and financial or resource constraints (Granger et al. 2022). The extent of which these contextual factors support or hinder the implementation and sustainability of health innovations is varied. However, what is common across health systems are persistent vulnerabilities including outdated services, inadequate access to comprehensive primary care, poor digital information systems and data governance, lack of effective means of spreading innovations, inadequate patient and public engagement and persisting health disparities (Granger et al. 2022). Nurses, trained in implementation science (‘nurse implementation scientists’) play a critical role in overcoming these vulnerabilities by virtue of the core competencies they possess.

A recent scoping review found nurse implementation scientists are usually PhD prepared and considered to have research expertise in implementation and/or improvement science. Nurse implementation scientists are distinguished leaders, mentors, researchers and practitioners, using their combined clinical experience and academic preparations to support the conduct and generation of new science (Granger et al. 2022). They offer knowledge, skills, qualifications and experience to lead health care improvements that health systems can learn from (i.e., familiarity with data sources, theory and literature) and apply over time to address areas of critical need, while considering changing health care contexts and health system constraints (Chambers 2020; Churruca et al. 2019; National Academies of Sciences, Engineering, and Medicine 2021; Tyczkowski and Reilly 2017).

### 2.3 Implementation Science Theories, Models, and Frameworks

Building improvement capacity within health systems should be informed by implementation science theories, models or frameworks (TMFs) (Table 2). TMFs provide a systematic structure for carrying out research, a basis for explaining how and why innovations work or not, contextual understanding across studies and evidence to support understanding of the mechanisms that facilitate or hinder the implementation, uptake and sustainability of innovations (Damschroder 2020; Mitchell et al. 2010; Smith, Li, and Rafferty 2020; Tabak et al. 2012). Research undertaken in complex, dynamic settings that lack adequate underpinning from a TMF may miss key information related to system‐wide processes, organisational factors and measures required for success (Davis and Beidas 2021).

**TABLE 2:** Implementation science theories, models and frameworks.

Category	Description	Examples
Process Models	Specify steps (stages, phases) Aim to describe and/or guide the process of KT Provides practical guidance in the planning and execution of KT	CIHR Model of Knowledge Translation, Knowledge‐to Action Framework, Iowa Model and Ottawa Model
Determinant frameworks	Specify types (classes or domains) of determinants and individual determinants, which act as barriers and enablers (independent variables) that influence implementation outcomes (dependent variables). Aim to understand and/or explain influences on implementation outcomes (i.e., predicting outcomes or interpreting outcomes retrospectively)	Promoting Action on Research Implementation in Health Services (PARIHS), Ecological Framework, Consolidated Framework for Implementation Research (CFIR) and Theoretical Domains Framework (TDF)
Implementation theories	Theories developed by IS researchers to provide understanding and/or explanation of aspects of implementation.	Organisational Readiness, Capacity‐Opportunities Motivation‐Behaviour (COM‐B) and Normalisation Process Theory (NPT)
Evaluation frameworks	Specify aspects of implementation that could be evaluated to determine implementation success	Reach, Effectiveness, Adoption, Implementation, Maintenance (RE‐AIM), 8 Outcomes for Implementation Research (Proctor) and PRECEDE‐PROCEED
Classic theories	Originating from fields external to IS which can be applied to provide understanding and/or explanation of aspects of implementation	Diffusion of Innovations, social cognitive theories, cognitive processes and decision‐making theories, and organisational theories

*Note:* Table adapted from: Mehta, T. G., Mahoney, J., Leppin, A. L., Stevens, K. R., Yousefi‐Nooraie, R., Pollock, B. H., Shelton, R. C., Dolor, R., Pincus, H., Patel, S., & Moore, J. B. (2021). Integrating dissemination and implementation sciences within Clinical and Translational Science Award programs to advance translational research: Recommendations to national and local leaders. Journal of Clinical and Translational Science, 5(1), e151. https://doi.org/10.1017/cts.2021.815.

Selecting an appropriate TMF to guide the research process requires expert knowledge of each TMF including its scope, purpose, constructs and measures. Implementation scientists have this expert knowledge and carefully consider these factors in relation to the setting and population under study, available resources and what stage of the implementation process the research is focused on.

### 2.4 Overview of the Issue

Nurse implementation scientists have much to offer, leading to roles that have expanded from academia into health systems where nurse implementation scientists generate new knowledge and implement evidence into practice (Allen et al. 2023; Birkhoff et al. 2020; Carter et al. 2020; Logsdon et al. 2017). However, there is limited literature providing practical examples of *how* nurse implementation scientists can make meaningful impacts within health systems (McNett et al. 2021; Ridge 2021). Literature that does provide such examples typically focus on nurse implementation scientists employed in large‐scale, urban, ‘research‐intensive’ settings with continuous access to funding, infrastructure and personnel to assist with research endeavours (Birkhoff et al. 2020; Hampton and Williams 2023; Jang, Weberg, and Dower 2018; Logsdon et al. 2017). Thus, there is a considerable gap offering tangible examples of how nurse implementation scientists can support smaller scale, rural health systems with fewer resources, like Health PEI. The aim of this paper is to demonstrate *how* nurse implementation scientists can support health system transformation by using Health PEI as a health system case exemplar. To support our discussion, we offer practical examples of ways nurse implementation scientists can support Health PEI in addressing key priorities outlined in the 2021–2024 strategic plan (Health PEI 2022). While this paper focuses on specific activities led by nurse implementation scientists within one health care system, in one Canadian province, many of the learnings and recommendations gleaned from this case exemplar can be adopted and scaled to meet the needs of health care systems experiencing similar challenges and vulnerabilities, worldwide. To further illustrate this global applicability, we mapped the nurse implementation scientist role within Health PEI to the NIRN's domains and competencies of implementation scientists.

## 3 Data Sources

Using Health PEI's 2021–2024 Strategic Plan as a case exemplar and framework for discussion, we highlight *how* nurse implementation scientists can support health system transformation. Health PEI's 2021–2024 Strategic Plan identifies four overarching goals relating to people, quality and safety, access and coordination, and innovation and efficiency (Health PEI 2022) (See Supplementary File 1). To achieve these goals, Health PEI has identified several key priority areas to focus on while recognising ongoing accomplishments each year in the Health PEI Annual Report (Health PEI 2023). We mapped selected key priorities outlined in Health PEI's current Strategic Plan with practical examples of *how* nurse implementation scientists can collaborate with Health PEI to address these priorities, achieve their goals and fulfil their mission of delivering inclusive, innovative and person‐centred care to all (Health PEI 2022).

### 3.1 Setting

#### 3.1.1 Prince Edward Island

Prince Edward Island (PEI) is Canada's smallest province with a population of over 170,000 residents who locals refer to as ‘Islanders’. From 2021 to 2022, PEI's population increased by just over 3.5% with a record number of immigrants (3400+) landing on PEI (PEI Statistics Bureau 2023). PEI's population is not only growing but also ageing. According to Health PEI's Annual Report (2023), the median age of those living on PEI is 42 years with approximately 20% of the population aged 65 years and older. Population growth and age have been increasing on PEI for several decades. The prevalence of chronic conditions such as diabetes, chronic obstructive pulmonary disease, high blood pressure and mood disorders are high on PEI compared to the rest of Canada (Health PEI 2023). As of June 2023, approximately 18% of Islanders were without a family doctor or nurse practitioner (NP) and this number is likely under‐reported as it only accounts for those registered on the Patient Registry (Spindle Strategy 2023). In addition to the number of Islanders awaiting a primary care provider, there are significant gaps in specialty health care services particularly in internal medicine and anaesthesiology. These service gaps result in a backed‐up and over‐burdened system, long wait times, and/or Islanders needing to seek care out of province (Spindle Strategy 2023). PEI is also unique in that nearly 50% of the province is farmland, and approximately 34% of Islanders live in rural areas, supporting the need to focus on rural health care throughout the province (PEI Statistics Bureau 2023). Consideration of PEI's health care operations, population, health demographics and critical service gaps are essential in understanding how to transform PEI's health system and ultimately the health and wellness of Islanders.

#### 3.1.2 Health PEI

Health PEI is responsible for the operation and delivery of publicly funded health care services in both acute care and community settings on PEI (Health PEI 2023). Health PEI is governed by The Health PEI Board who is accountable to the Minister of Health and Wellness. The Board is connected to the operational organisation, its achievements and conduct through the Chief Executive Officer of Health PEI (Health PEI 2022) (See Supplementary File 2). PEI has numerous health care facilities including two main referral hospitals, four community hospitals, one in‐patient psychiatric facility, one provincial palliative care programme, nine public long‐term care facilities, seven community mental health sites, five community addictions sites and one provincial addictions treatment facility. Additionally, PEI has numerous community health sites offering services in primary care, public health and home care (Health PEI 2022).

Health PEI has embedded nurse leaders throughout the organisation. For instance, former interim Chief Executive Officer (CEO) (now Special Advisor to CEO) and the Chief Nursing and Professional Practice Officer are both master's prepared registered nurses (RNs) (Government of Prince Edward Island 2024a). Health PEI's Board of Directors includes one retired RN, who also serves as Chair of Community Health Engagement for the Eastern region of PEI (Government of Prince Edward Island 2024b). In addition, nurse leaders are present throughout Health PEI facilities including those working on the front lines occupying informal leadership roles, and those in management and other formal leadership positions, such as nurse managers. External to Health PEI, nursing leadership is evident within The College of Registered Nurses & Midwives (CRNMPEI; https://www.crnpei.ca), PEI's regulatory body for RNs, nurse practitioners (NPs) and registered midwives in the province and the PEI Nurses' Union (PEINU; https://www.peinu.com), whose president serves to negotiate improved working conditions for all RNs and NPs on PEI. The University of Prince Edward Island (UPEI) and Holland College, PEI's main post‐secondary institutions, employ doctoral and master's prepared nurse academics and clinical nursing instructors to teach the future generation of RNs and licensed practical nurses (LPNs) and conduct nurse‐led research projects. Effective nursing leadership practices have critical implications for improved health care quality and patient outcomes (Cummings et al. 2021; Wong, Cummings, and Ducharme 2013). As nurses fulfil leadership positions from bedside to boardroom, it is crucial that health systems strategically employ nurse leaders who have experience and expertise required to fulfil the demands of such positions. Thus, we argue that health systems (including Health PEI) should capitalise on the opportunity to embed nursing leaders with implementation science expertise who have the requisite knowledge, skills and experience to lead critical research initiatives aimed at enhancing health care services, delivery and ultimately patient outcomes.

## 4 Findings

Our findings focus on selected key priorities in Health PEI's Strategic Plan accompanied by practical examples of the nurse implementation scientist role in achieving Health PEI's goals. An overview of these examples related to each key priority is provided in Table 3. In Table 4, we further mapped these examples to applicable core competencies and activities of the implementation scientist as described by the NIRN.

**TABLE 3:** Nurse implementation scientist role mapped to health PEI strategic goals and priorities.

Key priority	Nurse implementation scientist role
Strategic Goal #1: People *Establish a healthy, safe and high‐performing workplace that supports and develops our people*	
Attract and retain a skilled and high‐performing workforce for Health PEI	Gain an accurate understanding of Health PEIs workforce through collection of local data Understand context‐specific barriers and facilitators to recruitment and retention at an individual, unit and organisational level Conduct rigorous evaluations of current recruitment and retention innovations implemented within Health PEI and identify opportunities for adaptations or de‐implementation Engage with key stakeholders to co‐design new recruitment and retention innovations for implementation, ongoing monitoring and evaluation within Health PEI
Strategic Goal #2: Quality and Safety *Integrate quality and patient safety into the culture of the organisation*	
Create a psychologically and physically safe workplace where staff are supported by the appropriate resources, equipment, training and tools.	Proactively engage with front line health care providers to co‐design innovations addressing their specific needs Conduct rigorous evaluations on the implementation, uptake and outcomes of Health PEI employee wellness programmes Understand context‐specific barriers and facilitators to uptake of employee wellness programmes among health care providers within Health PEI
Embed understanding and prioritisation of quality and impacts on patient care throughout the organisation.	Embed nurse implementation scientists within Health PEI for continual and timely implementation and evaluation of innovations to meet changing population needs and priorities Assess organisational readiness for change including capacity, fit, resource availability, leverage and sustainability‐related factors gain an accurate understanding of population needs through collection of local data Identify and share key learnings from ongoing changes occurring within Health PEI and impacts on care quality
Strategic Goal #3: Access and Coordination *Provide quality, equitable and patient‐focuses care across the province*	
Increase access to primary care services and enhance delivery of care	Identify core components of successful primary care models including the circumstances and settings required for success by applying IS TMFs Understand the barriers and facilitators to the design, implementation and evaluation of primary care innovations Conduct a needs assessment to determine what innovations should be prioritised at an individual, unit and system level Evaluate the evidence and make recommendations for planning, implementation and evaluation of primary care innovations
Transition towards team‐based care to provide integrated and coordinated care	Establish improvement teams within Health PEI with embedded nurse implementation scientists to work collaboratively across settings and disciplines Lead and provide guidance/mentorship in conducting IS research across disciplines and settings to build capacity within Health PEI in selecting, designing, implementing and evaluating innovations to promote local ownership Collaborate with Health PEI and decision‐makers for rigorous planning and evaluations
Strategic Goal #4: Innovation and Efficiency *Develop new and innovative approaches to improve efficiency and utilisation of health care resources*	
Support the sustainability of the health system by building efficiencies across Health PEI	Strategically and intentionally plan for implementation, uptake, monitoring, evaluation and sustainability of innovations implemented within Health PEI Scale up and spread innovations across Health PEI Identify barriers and facilitators to sustaining innovations within Health PEI over time Test and refine innovations periodically, in the settings in which they are implemented Integrate principles of a LHS into Health PEI for a data‐driven, rapid response to continually changing evidence

**TABLE 4:** Example of implementation scientist core competencies and activities mapped to health PEI nurse implementation scientist role.

Implementation scientist core competencies and activities	Health PEI nurse implementation scientist role
*Key Priority: Attract and retain a skilled and high‐performing workforce for Health PEI*	
Assessing needs and assets^1^: Use data‐driven inquiry methods to support ‘discovery’ processes that holistically consider need, such as assessment data, stakeholder analysis, mapping of existing services, initiative inventory, etc	Gain an accurate understanding of Health PEIs workforce through collection of local data
Co‐design^1^: Support the co‐design and/or modification of specific implementation strategies based on resources and conditions present in the local context	Engage with key stakeholders to co‐design new recruitment and retention innovations for implementation, ongoing monitoring and evaluation within Health PEI
Tailoring support^1^: Continuously promote the adaptability of implementation strategies used by stakeholders	Conduct rigorous evaluations of current recruitment and retention innovations implemented within Health PEI and identify opportunities for adaptations or de‐implementation
Understanding context^2^: Assess the contextual fit of the proposed intervention(s)/approach(es) with the values, needs, skills and resources available in the service setting, including in specific subgroups, current political, funding and organisational landscape	Understand context‐specific barriers and facilitators to recruitment and retention at an individual, unit and organisational level
*Key Priority: Create a psychologically and physically safe workplace where staff are supported by the appropriate resources, equipment, training and tools*	
Co‐design^1^: Work with stakeholders to build a strong fit between intervention and implementation context before moving forward with implementation efforts	Proactively engage with front‐line health care providers to co‐design innovations addressing their specific needs
Conducting improvement cycles^2^: Promote the collection and use of data suitable to understand the differential impact of interventions and their implementation on different focus populations and communities	Conduct rigorous evaluations on the implementation, uptake and outcomes of Health PEI employee wellness programmes
Facilitation^2^: Support the continuous and systematic identification of barriers to implementation among different stakeholders	Understand context‐specific barriers and facilitators to uptake of employee wellness programmes among health care providers within Health PEI
*Key Priority: Embed understanding and prioritisation of quality and impacts on patient care throughout the organisation*.	
Brokering^1^: Position yourself as a bridge ‘in between’ people or groups that exist in a system and are vital for the success of an implementation	Embed nurse implementation scientists within Health PEI for continual and timely implementation and evaluation of innovations to meet changing population needs and priorities
Assessing needs and assets^2^: Support the identification of readily available and potential resources and assets to be utilised and leveraged in the implementation context	Assess organisational readiness for change including capacity, fit, resource availability, leverage and sustainability‐related factors
Assessing needs and assets^2^: Disaggregate available evidence and data and assess, consider and discuss needs that may exist for particular subpopulations (e.g., race, ethnicity, gender, socio‐economic status and geography)	Gain an accurate understanding of population needs through collection of local data
Facilitation^2^: Work with stakeholders to develop communication protocols designed to intentionally engage stakeholders, communicate progress and celebrate implementation success, report systemic barriers that are preventing or hindering implementation, report on actions taken to resolve or address implementation challenges, revisit past decisions and agreements periodically to ensure that solutions are still appropriate	Identify and share key learnings from ongoing changes occurring within Health PEI and impacts on care quality
*Key Priority: Increase access to primary care services and enhance delivery of care*	
Applying and integrating implementation TMFs, strategies and approaches^2^: Support the selection, application and integration of the range of implementation frameworks, approaches, tools and resources that are best suited for the local context of service and policy settings	Identify core components of successful primary care models including the circumstances and settings required for success by applying IS TMFs
Understanding context^2^: Assess the contextual fit of the proposed intervention(s)/approach(es) with the values, needs, skills and resources available in the service setting, including in specific subgroups	Understand the barriers and facilitators to the design, implementation and evaluation of primary care innovations
Assessing needs and assets^2^: Use data‐driven inquiry methods to support ‘discovery’ processes that holistically consider need, such as assessment data, stakeholder analysis, mapping of existing services, initiative inventory, etc.	Conduct a needs assessment to determine what innovations should be prioritised at an individual, unit and system level
Conducting improvement cycles^2^: Help to identify and gather relevant quantitative and qualitative data about the progress and quality of implementation activities and outcomes	Evaluate the evidence and make recommendations for planning, implementation and evaluation of primary care innovations
*Key Priority: Transition towards team‐based care to provide integrated and coordinated care*	
Brokering^1^: Develop and regularly convene implementation groups and teams with diverse stakeholders	Establish improvement teams within Health PEI with embedded nurse implementation scientists to work collaboratively across settings and disciplines
Co‐design^1^: Support collaborative implementation planning and co‐development of an implementation blueprint or plan involving all relevant stakeholders	Collaborate with Health PEI and decision‐makers for rigorous planning and evaluations
Building capacity^3^: Promote collaboration and partnerships as a way to build and expand capacities (e.g., through the use of community–academic partnerships, learning collaboratives, etc.)	Lead and provide guidance/mentorship in conducting IS research across disciplines and settings to build capacity within Health PEI in selecting, designing, implementing and evaluating innovations to promote local ownership
*Key Priority: Support the sustainability of the health system by building efficiencies across Health PEI*	
Applying and integrating implementation TMFs, strategies and approaches^2^: Include all relevant stakeholders in the selection, combination and co‐design of implementation strategies and approaches	Strategically and intentionally plan for implementation, uptake, monitoring, evaluation and sustainability of innovations implemented within Health PEI
Tailoring support^1^: Continuously promote the adaptability of implementation strategies used by stakeholders	Test and refine innovations periodically, in the settings in which they are implemented
Conducting improvement cycles^2^: Develop stakeholders' capacity to continuously assess and use data for decision‐making about the ongoing planning, implementation and outcomes of a programme or practice through modelling, instruction and coaching.	Integrate principles of a LHS into Health PEI for a data‐driven, rapid response to continually changing evidence
Building capacity^3^: Identify and help to change and/or implement organisational processes and structures required to develop capacity for implementation (e.g., human resources, technology).	Scale up and spread innovations across Health PEI
Building capacity^3^: Support stakeholders in identifying potential future external and internal challenges to implementation (related to, e.g., finances, policies or staffing) and to develop strategies for building sufficient capacity to meet these	Identify barriers and facilitators to sustaining innovations within Health PEI over time

*Note:* ^1^ = Domain 1: Co‐creation and engagement; ^2^ = Domain 2: Ongoing improvement; ^3^ = Domain 3: Sustaining change.

### 4.1 Strategic Goal #1: People

Establish a healthy, safe and high‐performing workplace that supports and develops our people

#### 4.1.1 Key Priority: Attract and Retain a Skilled and High‐Performing Workforce for Health PEI

Health sectors worldwide are under pressure to recruit and retain qualified workers (Morris et al. 2023). Health PEI is experiencing this pressure first‐hand, as the number of qualified health care providers throughout the province is insufficient to meet the growing needs of Islanders (Khandelwal 2024; Morse 2023b; Peachy et al. 2023; Spindle Strategy 2023). PEI has attempted to attract and retain health care providers using various strategies including focusing on international recruitment, changed equivalencies for Internationally Educated Nurses (IENs) and implementing a matching process for graduating students to externally posted roles to reduce the time it takes to fill these positions (Health PEI 2023). Additionally, a new medical school is under development at UPEI with plans for the first intake of students to begin in 2025, and a hope that more doctors will be enticed to train and practice on PEI in the future (Spindle Strategy 2023, 2024). Despite these efforts, significant shortages persist throughout the system. For instance, recent reports identified, as of January 2024, PEI has approximately 50 full‐time equivalent (FTE) positions for physicians that are vacant, with shortages most apparent in internal medicine, anaesthesiology and family practice (Spindle Strategy 2024). Additionally, it is projected that approximately 50 new FTE physicians will have to be hired into the health system by year 9 of UPEI's new medical school operations to meet medical education and clinical backfill needs (Spindle Strategy 2023, 2024). The PEINU reported approximately 350 vacant nursing positions within Health PEI as of November 2023, with unprecedented vacancy rates of approximately 30%–35% for RNs and NPs (Prince Edward Island Nurses Union 2024). These reports have offered recommendations for recruiting and retaining nurses and doctors, as it is apparent Health PEI's current efforts are not addressing this critical need. Recommendations include flexible employment options (i.e., full‐time, part‐time, short‐term/seasonal commitments), recognition and incentives for enhanced training and certifications, optimising workloads across disciplines (i.e., collaborative care models, expanded practice scopes), offering adequate training and psychological support and greater financial compensation (i.e., student debt forgiveness, introduction/increased payment for providing education/preceptors) (Prince Edward Island Nurses Union 2024; Spindle Strategy 2024). These recommendations are in alignment with evidence‐informed innovations that have been identified to attract and retain health care workers (Morris et al. 2023; Williamson, Burog, and Taylor 2022). However, simply identifying and implementing these innovations is not enough to improve recruitment and retention. It is essential to consider several factors that may facilitate or hinder the success of these innovations before, during and after implementation.

##### 4.1.1.1 Nurse Implementation Scientist Role

One factor that may serve as a barrier to successful recruitment and retention is that many organisations, including Health PEI, depend on the Canadian Institute for Health Information (CIHI) to understand the workforce in their jurisdiction. These data are often skewed to FTEs, and not actual workers needed to fill roles. Consequently, this leads to poor planning resulting in inadequate staffing ratios, qualifications and skills required to meet the needs of patients and their families. For instance, Health PEI's 2022–2023 Annual Report indicates there are 1847 nursing staff throughout the province. This number includes NPs, RNs, LPNs, resident care workers (RCWs) and personal care workers (PCWs) (Health PEI 2023). This number only reflects those working permanent, FTEs, excludes temporary and casual workers and does not reveal hours of work or effective productivity. Further, the variation in scope of practice among these disciplines impedes our ability to understand where the biggest need is in relation to nursing staff support which ultimately leads to poor recruitment and retention planning. This can be said for other disciplines with varying practice scopes and sub‐specialties. Planning errors like this directly impact patient care and provider outcomes because poor planning leads to poor evaluation (Tomblin Murphy et al. 2022). Rigorous evaluations are critical to understanding the effectiveness of innovations, programmes and policies. Nurse implementation scientists can help mitigate this issue by offering the skills and expertise required for local data collection, strategic planning, monitoring and evaluation of recruitment and retention innovations within Health PEI. Nurse implementation scientists recognise there is not a ‘one size fits all’ solution to recruiting and retaining health care workers. Solutions must be tailored to different disciplines, and different contexts, even within the same organisation, as they will each have unique needs. This aligns with a key principle of implementation science that it is not sufficient for evidence to simply exist. Implementation scientists have expertise with user‐centred design, making innovations useful, usable and desirable. Implementation scientists also recognise the critical importance of engaging stakeholders (i.e., health care providers) in designing, selecting and/or tailoring innovations and have the empirical knowledge and skills to work with key stakeholders in leading the design and execution of research projects (Mehta et al. 2021).

Another important consideration for recruitment and retention planning on PEI is the health and social impacts of the province's ageing population, growth in mental and behavioural health conditions and inadequate access to primary care. The implications of these issues will increasingly fall on health care providers and simply increasing the number of qualified providers is not enough to address these issues. Rather, increasing the number of providers in areas of critical need may be more beneficial to patient, provider and system outcomes (National Academies of Sciences, Engineering, and Medicine 2021). For example, there is a global need to increase the number of nurses in gerontology, mental and behavioural health and primary care (National Academies of Sciences, Engineering, and Medicine 2021). Considering PEI's health care context, these needs are particularly prevalent on PEI, and must be considered for recruitment and retention efforts to yield maximum benefits to Islanders (Morrison 2021). Another prominent contextual factor that must be considered regarding recruitment and retention on PEI is the rurality of the province. Rural health services are often modelled on urban services which may be counterproductive and threaten workforce stability. When service needs cannot be met by professionals in existing health service models, burnout and job dissatisfaction may occur (Abelsen et al. 2020). This is already occurring on PEI as evidenced by numerous physicians and nurses leaving their positions due to the demands imposed on them within Health PEI's current system (Campbell and Ryan 2023; Khandelwal 2024; Morse 2023a; Ryan 2023; Yarr 2024). Recruiting and retaining health care workers into a system that is not designed to context is a misuse of time and resources (Abelsen et al. 2020). Implementation scientists can engage with government and health authorities to overcome these challenges by virtue of their expertise in identifying contextual factors that influence adoption, implementation, maintenance and reach of health care innovations, including those related to recruitment and retention and health service models. This expertise is in direct alignment with another key principle of implementation science, that context matters, is multi‐level and must be explored to understand what works, for whom and in what circumstances (Braithwaite, Marks, and Taylor 2014; Braithwaite et al. 2018).

#### 4.1.2 Key Priority: Create a Psychologically and Physically Safe Workplace Where Staff Are Supported by the Appropriate Resources, Equipment, Training and Tools

Health care providers are essential to global health security. The nation's economy is dependent on a health care workforce that is adequately resourced, supported and renumerated, which was further reinforced by the COVID‐19 pandemic (Buchan, Catton, and Shaffer 2022; Eckert 2020). The responsibility for addressing health care provider's well‐being lies with the providers themselves and the systems, organisations and structures that support them. It has also been suggested that recruitment and retention are likely to be influenced by how an organisation's actions are perceived by potential workers (Morris et al. 2023). Health care workers have many options for employment, which has only been heightened by increasing labour shortages, and employees expect their employers will act ethically and responsibly in terms of employment practices. Indeed, health care organisations are increasingly recognising the strong linkage between employee well‐being and safe, high‐quality services (Morris et al. 2023). There is an urgent need for health systems to be reimagined to reduce increasing burdens on health care professionals, promote their health and well‐being and thus, their ability to provide care and achieve optimal patient outcomes. PEI requires a health system driven by the needs of patients and providers; and not by demands of increased efficiency, economic interests or political agendas (Søvold et al. 2021). Nurse implementation scientists are ideally positioned to bring this vision to fruition.

##### 4.1.2.1 Nurse Implementation Scientist Role

There are existing programmes and initiatives within Health PEI aimed at improving employee health and well‐being, such as a Provincial Employee Health section on Health PEI's website, and creation of a Provincial Wellness Committee (Health PEI 2023). However, there is insufficient evidence available to understand the impact of these innovations and their relationship to system factors. Without this understanding, it is difficult to fully understand the severity of the issues health care workers on PEI are facing and limits Health PEI's ability to address them. Once again, implementation scientists recognise it is not sufficient that evidence simply exists. For evidence to be effectively implemented and utilised in practice, nurse implementation scientists should be consulted to support the planning, implementation and sustainability of these evidence‐informed innovations by assessing barriers and drivers to their success. Furthermore, nurse implementation scientists appreciate that implementation science and practices are team endeavours, and those who are most likely to be impacted by the innovation must be consulted at various time points for the innovation to be successfully translated into practice (Mehta et al. 2021). For example, a nurse implementation scientist would ensure front line health care workers are proactively engaged in designing a diversity of resources that they perceive to be valuable in fostering their well‐being rather than having such resources imposed on them (National Academies of Sciences, Engineering, and Medicine 2021). This engagement, or *co‐production*, would ensure innovations are contextually relevant, focus on staff needs and ensure diverse perspectives are considered (Wehrens 2014). Furthermore, this engagement would glean insight into what outcome measures should be evaluated, which is critical to understanding whether an innovation is effective, and worth the investment of time, finances and resources (Morris et al. 2023).

### 4.2 Strategic Goal #2: Quality and Safety

Integrate quality and patient safety into the culture of the organization.

#### 4.2.1 Key Priority: Embed Understanding and Prioritisation of Quality and Impacts on Patient Care Throughout the Organisation

The 2021 PEI Chief Public Health Officer's Report states a key action for improving the quality of health and well‐being of Islanders is that ‘decisions, policies, and programs in PEI must be informed by evidence and should ensure that basic needs like housing, food security, transportation, education, and employment opportunities are met. Systemic discrimination and stigma lead to health inequities and must be addressed’ (Morrison 2021). Overcoming these issues in complex systems, like health care, is challenging, as there are multiple interacting and interdependent factors, high levels of variability and instability and simplistic ‘cause and effect’ or ‘command and control’ logic does not apply (Braithwaite et al. 2018; Melder et al. 2020). Furthermore, research on quality and safety innovations often do not adequately account for characteristics of the evidence (i.e., quality), complex contexts or facilitation strategies (Braithwaite, Marks, and Taylor 2014; Braithwaite et al. 2018). This understanding requires systematic, continuous, data‐driven and rigorous processes of assessment, innovation, implementation, evaluation and translation of the evidence or best practices into tangible strategies or policies for improving health (National Academies of Sciences, Engineering, and Medicine 2021). Nurse implementation scientists are ideally situated to lead these efforts.

##### 4.2.1.1 Nurse Implementation Scientist Role

A systematic review identified key success factors to improve care quality and patient safety within health organisations (Braithwaite, Marks, and Taylor 2014). These include preparing for change, capacity for implementation (i.e., are there enough people with necessary skills to implement the innovation and is the setting receptive to change?), fit of the innovation, resource availability (i.e., human and financial), leverage, sustainability and desirable implementation features (i.e., planning, project management, communication, collaboration, monitoring, evaluation and tailoring to local context) (Braithwaite, Marks, and Taylor 2014). Reinforcing and opening eyes to implementation science by an expert implementation specialist was also found to be a success factor, further emphasising the need for trained implementation scientists (Jeffs et al. 2023; Wilson and Kislov 2022). To successfully integrate evidence into practice for meaningful change, not only do we need evidence to understand what works, but we also need clinical leaders, like nurse implementation scientists, with the skills to lead teams to determine what elements facilitate or hinder effectiveness. Health care providers want to know how their practical problems can be addressed by selecting and designing innovations and determining how to make the innovation work in practice settings with numerous obstacles, while rapidly evaluating its success. These questions can be answered through the application of implementation science methods if adequate expertise and resources are available to support them (Wilson and Kislov 2022).

Another way nurse implementation scientists can foster embedded understanding and prioritisation of system and population needs is through embedded research where nurse implementation scientists work across different sites and settings within the health system and work with relevant partners to answer research questions that match their priorities. Embedding nurse implementation scientists across sites and settings builds system capacity and capability to support primary, local data collection and foster organisational readiness for change, both of which are key elements to the successful implementation and adoption of health care innovations (Wilson and Kislov 2022). Another major benefit of embedded research is instead of one‐off studies, evaluation and research are built into implementation efforts for sequential, timely and rapid learning (Beidas et al. 2022). The COVID‐19 pandemic highlighted the importance of being able to rapidly generate and incorporate research evidence to support policy and practice decisions during unprecedented events with a focus on quality and safety (Cassidy, Flynn, and Shuman 2021). Implementation scientists pivoted to use rapid implementation science methods to meet the needs of different settings and populations, exemplifying how to offer their skillset to health systems in times of critical need (Beidas et al. 2022). Not only can embedded nurse implementation scientists identify and implement effective innovations, they can also identify key learnings from ongoing changes within health systems so quality and safety are maintained, and generalisable knowledge is gleaned to transform other organisations and systems (Chambers 2020). On PEI, this knowledge could position Health PEI to be a leader in rural health system transformation. Another benefit of embedded nurse implementation scientists is that efforts to introduce change now sit at the intersection of implementers' (i.e., Health PEI, Minister of Health and Wellness) appreciation of change and their capacity to influence context and researchers' (i.e., nurse implementation scientists) knowledge and expertise in empirical methods (Churruca et al. 2019). Embedded nurse implementation scientists will provide implementers with feedback and findings more quickly so adaptations can be made accordingly in a timely manner.

Furthermore, as new evidence emerges and additional challenges arise in health care, a range of new approaches will be developed as an attempt to improve the quality of care delivery and outcomes (Chambers 2020). Discerning what evidence is reputable or what approach will yield the most benefit to patients, providers and administrators is a priority. Nurse implementation scientists have the experience and knowledge to address these concerns and lead studies to distinguish whether insufficient benefits to health result from ineffective innovations or from effective innovations that were ineffectively implemented (Chambers 2020). This understanding is important when considering the quality and impacts of health innovations in addition to the scale and spread of innovations throughout the system.

### 4.3 Strategic Goal #3: Access and Coordination

Provide quality, equitable and patient‐focused care across the province.

#### 4.3.1 Key Priority: Increase Access to Primary Care Services and Enhance Delivery of Care

Health systems with strong primary care services are associated with greater equity, lower mortality and fewer disparities in measures of health outcomes and health care use (Glazier 2023). In recognition of this key priority area, PEI has made investments in community health centres across the province and are piloting new integrated care centres known as ‘Patient Medical Homes’ (Spindle Strategy 2024). In rural settings like PEI, health care providers often work in isolation without immediate access to specialist support, have limited resources and may feel overwhelmed and overburdened, further emphasising the need for collaborative, primary care practices (Abelsen et al. 2020). Another way to enhance the delivery of primary care services is to optimise and expand health care provider's scope of practice within primary care, especially in rural settings. For instance, evidence suggests that RNs can provide superior diabetes care and are effective case managers for patients with complex needs (Glazier 2023). Similarly, pharmacists can manage patients with hypertension and other cardiovascular disease risks. Nurse practitioners can autonomously diagnose and treat illnesses, order and interpret tests and perform advanced medical procedures (Glazier 2023). This concept, known as task shifting, has been endorsed for decades by one of the major global policy summits, which has also called for creation of policies and mechanisms to optimise skills‐needs mix (World Health Organization 2007). While these actions may bring forth improvements to primary health care services, their potential has not yet been achieved due to piecemeal planning, lack of resources and lack of education so that providers better understand integrated, collaborative primary care models and regulations (Glazier 2023). Indeed, collaborative care is central to workforce and clinical service planning with substantial and sustained impacts on primary care and outcomes (Peachy et al. 2023). However, it cannot be understated that innovations must be grounded in evidence informed decisions and undergo continuous evaluations to ensure the greatest success and impact (Health Canada 2024).

##### 4.3.1.1 Nurse Implementation Scientist Role

Nurse implementation scientists possess the knowledge and skills to overcome challenges related to primary care services through implementation science research. It was reported, as of February 2024, a dozen Patient Medical Homes in PEI are up and running or in the developing stages and an additional 17 are in development (Spindle Strategy 2024). It is expected these collaborative care practices will make it more attractive for family physicians, specialists and other health care workers to practice on PEI (Spindle Strategy 2024). However, the province must proceed with caution in establishing these patient medical homes to ensure they do lead to their intended outcome of improved care access and delivery. One way to ensure this outcome is achieved is delegating a nurse implementation scientist to examine what the core components of a patient medical home are, under what circumstances and in what settings are patient medical homes adopted, implemented well and maintained, what patient outcomes improve because of patient medical homes and what are the most cost‐effective implementation strategies for putting patient medical homes in place? Implementation science TMFs can be applied to investigate these important questions ensuring that not only the innovation (patient medical homes) is evidence‐informed, but that the implementation, adoption and evaluation processes are as well (Holtrop, Rabin, and Glasgow 2018).

Nurse implementation scientists are also essential for conducting research that is needed to facilitate and support expanded practice roles. For instance, although high‐level policy declarations call for governments to create an enabling policy environment for training and accreditation, research is needed to identify how to revise practice roles, shift tasks and measure effects (Geng, Moshabela, and Alonge 2023; World Health Organization 2007). Additionally, nurse implementation scientists understand contextual factors will likely influence the degree to which health care providers can optimise their scope of practice, including policies and regulations. Additional considerations may include costs associated with expanded practice scopes, impacts on workflow and unintended consequences or outcomes. This recognition by nurse implementation scientists should prompt implementers (i.e., Health PEI), to consider what multi‐level changes are required for primary care providers to maximise their scope of practice, what may help or hinder that process and how patient, provider and system outcomes can be feasibly measured.

#### 4.3.2 Key Priority: Transition Towards Team‐Based Care to Provide Integrated and Coordinated Care

A recent report detailing the current state of health care services on PEI and future predictions suggests that key to addressing the health and social services needs of Islanders will require revisioning Health PEI's current care delivery model and that collaborative, team‐based care may address many, if not all, the challenges faced in PEI (Peachy et al. 2023). To ensure successful implementation and adoption of a new collaborative model of care, programmatic, policy and funding opportunities must be leveraged by health systems, government, educators and community stakeholders (National Academies of Sciences, Engineering, and Medicine 2021). When health care providers, researchers, administrators and government work collaboratively, to the full breadth of their scope and expertise, enhanced care, lower health care costs, improved working environments and reduced clinician workloads across the board will be achieved.

##### 4.3.2.1 Nurse Implementation Scientist Role

There is a need for PEI to build and strengthen collaborative partnerships with government and implementation scientists. This will ensure instant access to the most up‐to‐date evidence and support the design, implementation and evaluation of policy and practice change innovations. In collaboration with health system partners, nurse implementation scientists can scale up and spread innovations that will address the changing needs of patients, health care providers and the health system (Cassidy et al. 2022). This kind of collaboration would reduce inefficiencies, costs and resources required for isolated research projects that are often implemented, but fail to be sustained, or may be unnecessary and potentially harmful due to inadequate planning and collaboration among key stakeholders.

Improvement teams have been suggested as one way to foster collaborations within complex, multi‐level health systems. These teams are often comprised of front line providers and staff working collaboratively with researchers (i.e., nurse implementation scientists) to plan and test improvements in their unique health care setting, thereby promoting local ownership of the change in practice (Kothari and Wathen 2017; Leeman et al. 2021). Research has shown that individuals are more likely to adopt and implement innovations when they view themselves as the owners of the innovation and are able to shape them to fit local needs and preferences. Local ownership has also been found as essential to sustaining innovations over time (Leeman et al. 2021). Implementation scientists should be part of improvement teams, providing expertise in monitoring implementation and adapting innovations over time in response to changes such as new regulations, new funding streams or changes in staffing or the population served (Leeman et al. 2021). As health systems and environments are variable and unpredictable, this expertise is essential to meet the ever‐changing needs of the health system, staff and patients. Importantly, regardless of whether financial and professional barriers are addressed, evidence will be required to understand what capacity PEI has to build strong, collaborative teams and better understand the barriers and facilitators to the successful implementation and evaluation of programmes and policies to support collaborative care centres. Nurse implementation scientists are integral to this understanding.

### 4.4 Strategic Goal #4: Innovation and Efficiency

Develop new and innovative approaches to improve efficiency and utilization of health care resources.

#### 4.4.1 Key Priority: Support the Sustainability of the Health System by Building Efficiencies Across Health PEI

Successful uptake of innovations into health care practice requires consideration of multiple system levels, carefully planned implementation strategies and the ability to adapt elements of evidence‐informed innovations to fit complex contexts (Flynn et al. 2021; Shelton, Cooper, and Stirman 2018). Transforming an efficient and sustainable health system requires strategic planning for ongoing monitoring and evaluation. Processes to support sustainability must be established during the planning process and anchored at various stages for ongoing monitoring (Braithwaite et al. 2018).

Sustainable and efficient health systems require innovations delivered at a scale to yield population health improvements. The sustainability of evidence‐informed innovations is known to be one of the ‘most significant translational research problems of our time’ (Proctor et al. 2015). One way to sustain evidence‐informed innovations is through scale up and spread. Scaling up refers to the process of ‘expanding or replicating pilot or small‐scale projects to reach more people and/or broaden the effectiveness of an innovation’ (World Health Organization 2016). The scale up process applies a systematic approach to support the adoption and implementation of innovations that have demonstrated an effect at the local level, to regional, national or international levels by addressing the system and infrastructure issues arising during full‐scale implementation (Ben Charif et al. 2017; Greenhalgh and Papoutsi 2019). Scale up requires collaborations with policymakers, end users and researchers to enhance dissemination, adoption, implementation and sustainability throughout an entire system to achieve greater health impacts.

##### 4.4.1.1 Nurse Implementation Scientist Role

Nurse implementation scientists are ideally positioned to foster collaborations across disciplines with relevant end users and decision‐makers to scale up and spread innovations. They are equipped to support sustainability efforts by conducting contextual assessments to understand the multi‐level factors that may influence the long‐term delivery and impact of an innovation, to inform planning and help address and reduce barriers to sustainability. Nurse implementation scientists have expertise in applying TMFs to these assessments to help provide shared governance and guidance between researchers, practitioners, policy and decision‐makers. For example, a nurse implementation scientist could apply the Integrated Sustainability Framework as a guide to identify multilevel factors that have been commonly associated with sustainability across different settings, contexts and populations (Shelton, Cooper, and Stirman 2018; Wolfenden et al. 2023). These factors include outer contextual or policy‐related factors (e.g., the broader sociopolitical context, existing policies, funding environment, external partnerships with organisations/stakeholders); inner contextual or organisational‐level factors (e.g., resources and internal funding, leadership, presence of programme champions, organisational readiness and support, staff stability; alignment of internal practices/policies; culture); implementation processes (e.g., ongoing training, planning for sustainability, stakeholder engagement; coaching/feedback, capacity building); characteristics of the innovation (e.g., adaptability, benefits, fit with setting and population); characteristics of implementers (e.g., skills, attitudes, motivations and role clarity); and characteristics of populations (e.g., literacy, language, experiences of stigma, mistrust, discrimination and other social characteristics) (Shelton, Cooper, and Stirman 2018).

Importantly, nurse implementation scientists acknowledge that sustainability should not be conceptualised as a static end goal. Sustainability is dynamic, and this recognition allows for capacity building and planned adaptations in response to changing evidence, population needs and varying complex contexts (Wolfenden et al. 2023). The Dynamic Sustainability Framework aligns well with this recognition by proposing that innovations should be tested and refined in the settings in which they are implemented so continual learning about the innovation can be achieved (Chambers, Glasgow, and Stange 2013). Tracking, monitoring and evaluating what implications these adaptations have on sustainability, in addition to health outcomes and costs are critical tasks which can be optimally undertaken by nurse implementation scientists.

The COVID‐19 pandemic presented an opportunity to explore how health systems adapt under rapid and constant change, gleaning insight that is valuable for informing the development of resilient, efficient and sustainable health systems (Cassidy et al. 2022). For example, a learning health system (LHS) has been proposed as an ideal structure to inform a data‐driven response to rapidly changing evidence on a continual basis and incorporate lessons learned across the health system (Cassidy et al. 2022; Friedman et al. 2015; Lavis et al. 2018; Reid 2016). This structure accelerates the process of integrating the most up‐to‐date research into real‐world practice (Friedman et al. 2015; Lavis et al. 2018; Reid 2016). As PEI works towards transforming their health system with innovative, efficient and sustainable approaches, a LHS could be one solution worth exploring further. A LHS requires ongoing implementation, spread, scale and sustainment of new innovations and rigorous evaluations of intended (or unintended) outcomes, all of which can be achieved through implementation science research and led by a nurse implementation scientist (Flynn et al. 2021; Shelton, Cooper, and Stirman 2018). While PEI has identified key priorities and potential actions to address them, the sheer volume and urgency of these actions poses the challenge of knowing where to start. A LHS with embedded implementation science expertise may benefit Health PEI, and others, by accelerating the most up‐to‐date research and innovations into real‐world practice.

## 5 Discussion

### 5.1 Implications for Research, Policy and Nursing Practice

It is clear PEI's health system is under a tremendous amount of strain with negative implications for patients, providers and the system overall. While the province has developed a comprehensive Strategic Plan, with clear goals, priorities and recommendations, improving health care services and outcomes for Islanders continues to fall short. We argue that Health PEI ought to embrace implementation science to promote the systematic uptake of innovations into practice, to ultimately improve the quality and effectiveness of health service delivery and patient care, and that nurse implementation scientists are ideally positioned to lead these efforts (Boehm, Stolldorf, and Jeffery 2020; Carter et al. 2020; Flynn et al. 2021; Hampton and Williams 2023; Jang, Weberg, and Dower 2018). This discussion highlights important implications for research, policy and nursing practice within Health PEI and beyond.

Nursing has been instrumental in leading efforts to bridge the research‐to‐practice gap in health care through research, theory development, clinical exemplars, intensive evidence‐informed training programmes and production of a plethora of resources (Birkhoff et al. 2020; Boehm, Stolldorf, and Jeffery 2020; Carter et al. 2020; Jang, Weberg, and Dower 2018). Nursing has also contributed to the growing body of inter and intra‐professional science dedicated to building knowledge about successful processes for evidence adoption, including identification of factors that influence, delay or accelerate the successful and sustained implementation of evidence into routine clinical practices (Tucker et al. 2021). Health care teams require both scientists and clinicians to lead evidence‐informed implementation and improvement efforts. Doctor of Philosophy (PhD) in Nursing and Doctor of Nursing Practice (DNP) programmes prepare nurses to carry out this important work and positions nursing to curate leaders with expertise in evidence‐informed clinical practices and application of implementation science (McNett et al. 2021; Tyczkowski and Reilly 2017). However, the slow growth in doctoral‐prepared nurses is a major concern for the profession and population health, as it is these nurses who serve as faculty at many universities, systematically studying issues related to health and health care and are needed to educate and prepare the next generation of nurses (Broome and Fairman 2018; Fairman et al. 2021). Furthermore, many joint nurse‐scientist roles pertain to positions adjoining clinical practice and teaching, rather than joint appointments between schools and health systems. The joint nurse‐scientist role offers a new model of research collaboration across settings. In health systems, the nurse‐scientist facilitates the scholarly work of others and has unique access to an array of data sources and a dynamic, real‐world ‘clinical laboratory’ to conduct research. In the academic setting, the nurse‐scientist receives and delivers ongoing mentorship and is part of a community of scholars, where research productivity and collaboration is expected. The joint role enables doctoral‐prepared faculty to influence the scholarly work of health system providers and collaborate on health system initiatives while advancing their programmes of research (Carter et al. 2020). While research has identified important outcomes related to the joint nurse‐scientist role between schools and health systems including rigorous and timely conduct of joint research, rapid translation of evidence into practice, opportunities to enhance and support scholarly activities among front line care providers and improved visibility and valuation of the doctoral‐prepared nurse, more research is needed to fully understand the effectiveness of joint nurse‐scientist roles within health systems and their impact on patient, provider, population and cost‐related outcomes, particularly in rural settings (Carter et al. 2020).

Applying research into health care practice is not only a technical matter of knowledge translation and exchange, but also a multifaceted political challenge (Bullock et al. 2021; Liverani, Hawkins, and Parkhurst 2013; Windle et al. 2023). In implementation science, a policy can be ‘the thing’ (i.e., evidence‐informed innovation) that is central to the research study, or it can be viewed as a contextual factor that may influence the implementation of an evidence‐informed innovation (Curran 2020). Regardless of how a policy is conceptualised, implementation science research can glean important policy‐related insights within health care. For instance, when a policy is conceptualised as the evidence‐informed innovation, implementation science research could explore *how* research evidence can be effectively communicated to policymakers and integrated into policymaking processes, test the effects of policy dissemination strategies or determine the effect of a policy on health outcomes. On the other hand, when a policy is conceptualised as a contextual factor, implementation science research could explore the effect of a policy on the implementation or effectiveness of an evidence‐informed innovation. Another way nurse implementation scientists could benefit government and policy is through research focusing on dismantling health inequities. For example, an implementation science study could focus on the adoption and implementation of social and economic policies (i.e., housing, education) that shape determinants of health and dismantle policies that reinforce other systems of oppression (Brownson et al. 2022). Additionally, many policymakers (and members of the public), are not knowledgeable about health inequities or their causes, and often have conflicting values and priorities resulting in challenging sociopolitical contexts in which policies are made and implemented (Denis, Usher, and Préval 2022; Gollust et al. 2020). In turn, this can lead to implicit biases, negative attitudes and poor messaging about health issues that are not evidence informed. This further supports the need for nurse implementation scientists with expertise on the importance of thoughtful message tailoring, dissemination and knowledge of clinical practice issues (Gollust et al. 2020; McHugh et al. 2022, 2023). Despite the recognition that political influences must be considered for successful implementation science research, further research is needed in this area. Health PEI may benefit from embedding a nurse implementation scientist to Health PEI to explore policy‐related factors impacting the Island's health system.

### 5.2 Global Relevance

Leadership and governance, products and services, health information systems and the health care workforce, are key dimensions of health care systems, globally. While contextual factors may determine the extent in which these dimensions impact and influence health care systems, the ways in which nurse implementation scientists can support these key dimensions are applicable across different settings and system structures. In addition to the universally applicable key dimensions of health care systems, persistent vulnerabilities including outdated services, inadequate access to comprehensive primary care, poor digital information systems and data governance, lack of effective means of spreading innovations, inadequate patient and public engagement and persisting health disparities are prevalent among health systems worldwide (Granger et al. 2022, Kim et al. 2024). Nurse implementation scientists play a critical role in overcoming these vulnerabilities and many of the learnings and recommendations gleaned from this discussion can be adopted and scaled to meet the needs of health care systems experiencing similar challenges and vulnerabilities. Furthermore, nurse implementation scientists can partner with other health system leaders to engage in reciprocal learning about the barriers and facilitators to successful implementation and uptake of health innovations in other settings (Kim et al. 2024) This reciprocal learning could promote the uptake of evidence‐informed innovations across settings, reduce waste and inefficiencies and ultimately improve outcomes to a broader population.

Nevertheless, despite the universal dimensions and vulnerabilities present in most health care systems, political and financial factors have a major role in determining how health care systems function and whether the nurse implementation scientist role could be supported. As Allen et al. (2023) suggests, the nurse implementation scientist role can be challenging to implement as not all health care system leaders have the same priorities, budgets, resources or understanding of the role. However, we argue that due to the increasing complexity of health care and health care service delivery, health care system leaders must be willing to endorse evidence‐informed and research‐focused infrastructures that support nurse implementation scientists to improve health care practices and outcomes and meet organisational goals. Indeed, nurse implementation scientists are well versed in strategically and intentionally planning the continuous monitoring, refinement, scale and spread of innovations over time in complex, rapidly evolving contexts. This has already been achieved in health systems across the globe that have embraced the nurse implementation scientist role (Birkhoff et al. 2020; Kim et al. 2024; Russell‐Babin et al. 2023; Theobald et al. 2018).

## 6 Strengths and Limitations

A key strength of this work is the identification of practical examples and recommendations of the nurse implementation scientist role mapped to a real‐world health system's Strategic Plan and priorities. While the focus on one, specific health system suggests our recommendations may not be applicable to other contexts with different strategic priorities, they do offer guidance and exemplars relevant to health systems in similar contexts, with similar populations and needs. Furthermore, the universal dimensions and vulnerabilities among health systems worldwide in addition to the universally applicable implementation science approaches to health system transformation and the core competencies of implementation scientists suggest our recommendations have broad applicability. We recognise health care systems are complex and would be remised in suggesting nurse implementation scientists can address all these complexities. This further emphasises the importance of collaboration across multiple levels of partners within and outside health systems, like the joint nurse‐scientist role. Additionally, it is outside the scope of this paper to comment on how nurse implementation scientists can address *all* the key priority areas of Health PEI's Strategic Plan. Nevertheless, we provide evidence of how we can address some prominent key priorities as a starting point for embedding nurse implementation scientists into Health PEI and beyond.

## 7 Conclusion

PEI's unique context serves as an ideal setting for implementation science research. Examining the implementation of evidence‐informed innovations in a unique, rural setting will glean insight into contextual variations that can broaden considerations of how implementation science can optimise health services and delivery within health system constraints in other contexts (Geng, Moshabela, and Alonge 2023). Embedding nurse implementation scientists into PEI's health system to lead initiatives outlined in this paper would position the province to be a leader in innovative health system transformations in Atlantic Canada. The knowledge and skillset of nurse implementation scientists will not only benefit nursing, but other health sectors and disciplines. However, to fully embrace the role, collaboration and buy‐in from health system leaders is essential, as is appropriate resourcing and compensation. Overall, this work identifies how implementation science and nurse implementation scientists can lead health system transformations in a rigorous, evidence‐informed way, to improve health outcomes for patients, providers and populations.

## Author Contributions

A.C. and C.C.: Made substantial contributions to conception and design, or acquisition of data, or analysis and interpretation of data; Involved in drafting the manuscript or revising it critically for important intellectual content; Given final approval of the version to be published. Each author should have participated sufficiently in the work to take public responsibility for appropriate portions of the content; Agreed to be accountable for all aspects of the work in ensuring that questions related to the accuracy or integrity of any part of the work are appropriately investigated and resolved.

## Conflicts of Interest

The authors declare no conflicts of interest.

### Peer Review

The peer review history for this article is available at https://www.webofscience.com/api/gateway/wos/peer‐review/10.1111/jan.16651.

## Supporting information


Data S1.

Data S2.

## Data Availability

Data sharing not applicable to this article as no datasets were generated or analysed during the current study.
