# Supplementary figures and images for: The Role of Nurse Implementation Scientists in Leading Health System Transformation in Atlantic Canada and Beyond: A Discussion Paper
Source: J Adv Nurs. 2024 Dec 6;81(6):3338–61. doi: 10.1111/jan.16651 (PMC12080080; doi:10.1111/jan.16651)

**Supplementary File 1**

Health PEI Strategic Plan Overarching Goals


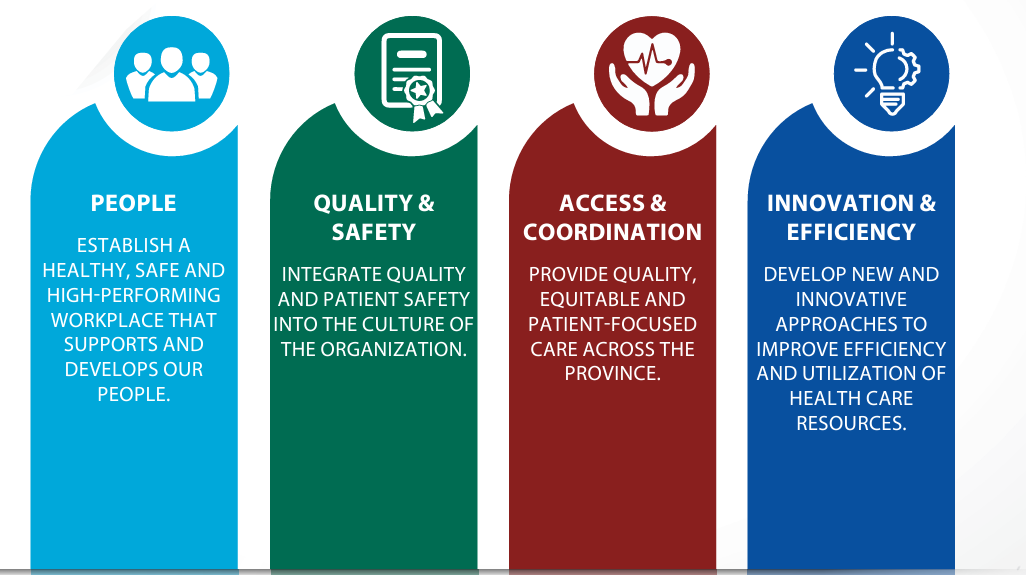

Supplement: Supplementary file 1 — Data S1. [file JAN-81-3338-s001.docx]

**Supplementary File 2**

Health PEI Organizational Structure


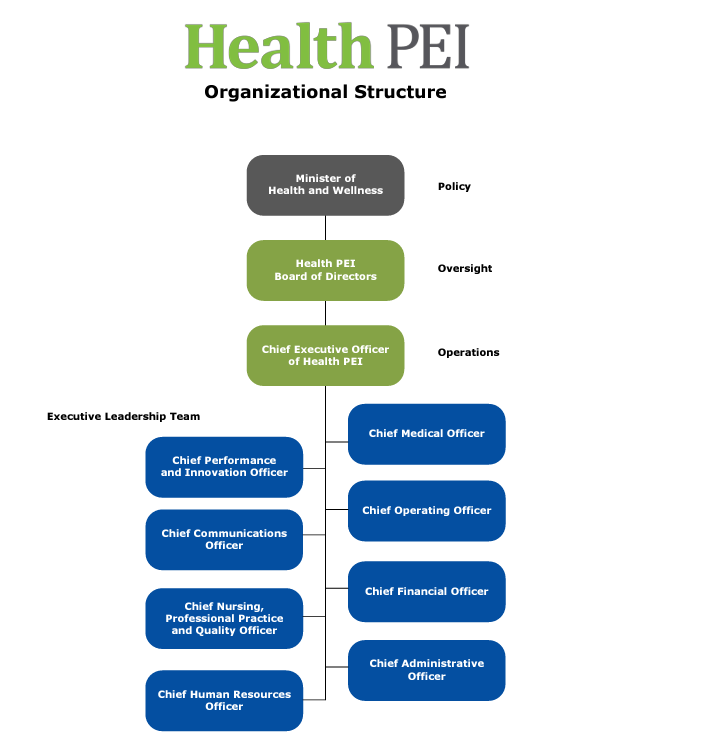

Supplement: Supplementary file 2 — Data S2. [file JAN-81-3338-s002.docx]
